# Whole-genome analyses reveal gene content differences between nontypeable *Haemophilus influenzae* isolates from chronic obstructive pulmonary disease compared to other clinical phenotypes

**DOI:** 10.1099/mgen.0.000405

**Published:** 2020-07-24

**Authors:** Rajendra KC, Kelvin W.C. Leong, Nicholas M. Harkness, Julia Lachowicz, Sanjay S. Gautam, Louise A. Cooley, Belinda McEwan, Steve Petrovski, Gunasegaran Karupiah, Ronan F. O'Toole

**Affiliations:** ^1^​ Tasmanian School of Medicine, College of Health and Medicine, University of Tasmania, Tasmania, Australia; ^2^​ Department of Pharmacy and Biomedical Sciences, School of Molecular Sciences, College of Science, Health and Engineering, La Trobe University, Victoria, Australia; ^3^​ Department of Respiratory and Sleep Medicine, Royal Hobart Hospital, Tasmania, Australia; ^4^​ Department of Microbiology and Infectious Diseases, Royal Hobart Hospital, Tasmania, Australia; ^5^​ Department of Physiology, Anatomy and Microbiology, School of Life Sciences, La Trobe University, Victoria, Australia

**Keywords:** chronic obstructive pulmonary disease, nontypeable *Haemophilus influenzae*, whole-genome sequencing, pan-genome-wide association studies

## Abstract

Nontypeable *Haemophilus influenzae* (NTHi) colonizes human upper respiratory airways and plays a key role in the course and pathogenesis of acute exacerbations of chronic obstructive pulmonary disease (COPD). Currently, it is not possible to distinguish COPD isolates of NTHi from other clinical isolates of NTHi using conventional genotyping methods. Here, we analysed the core and accessory genome of 568 NTHi isolates, including 40 newly sequenced isolates, to look for genetic distinctions between NTHi isolates from COPD with respect to other illnesses, including otitis media, meningitis and pneumonia. Phylogenies based on polymorphic sites in the core-genome did not show discrimination between NTHi strains collected from different clinical phenotypes. However, pan-genome-wide association studies identified 79 unique NTHi accessory genes that were significantly associated with COPD. Furthermore, many of the COPD-related NTHi genes have known or predicted roles in virulence, transmembrane transport of metal ions and nutrients, cellular respiration and maintenance of redox homeostasis. This indicates that specific genes may be required by NTHi for its survival or virulence in the COPD lung. These results advance our understanding of the pathogenesis of NTHi infection in COPD lungs.

## Data Summary

Sequence read files for all 40 isolates sequenced in this work have been deposited in National Center for Biotechnology Information (NCBI) Sequence Read Archive (SRA) and are accessible through NCBI BioSample and SRA accession numbers SAMN13942196 and SRS6166046, respectively.

Two supplementary figures, five datasets and a fasta file of non-redundant pan-gene sequences are available on Figshare (https://doi.org/10.6084/m9.figshare.12545957.v1).

Dataset 1. This dataset contains information on the clinical source and geographical origin of the 568 isolates of nontypeable *Haemophilus influenza* (NTHi) included in this study. The dataset also contains the clade number and multilocus sequence typing allelic profile of each isolate. The 40 NTHi isolates newly sequenced for this study are boxed for easy identification.

Dataset 2. This dataset contains data on pan genes as identified by Roary from the analysis of 568 NTHi genomes. The dataset provides information regarding gene annotation and the pan gene presence and absence profile of the 568 NTHi isolates.

Dataset 3. This dataset lists the names of genes that were enriched in isolates of a particular clade. The genes enriched in each clade are provided in separate worksheets.

Dataset 4. This dataset lists the genes associated with chronic obstructive pulmonary disease (COPD) strains of NTHi. This first worksheet lists the 680 genes that were found to be over- and under-represented in COPD strains. The second worksheet lists the 226 genes that were over-represented (odds ratio ≥2) in COPD strains. The third worksheet lists the 145 genes that were significantly associated with COPD strains. The fourth worksheet lists the 122 COPD-associated genes that were found to be significant after 1000 random permutations of the phenotypic data. The fifth worksheet lists the 79 genes that were unique (variants of the same genes were excluded) and were significantly associated with the COPD strains of NTHi.

Dataset 5. This dataset contains information on the functional annotation and classification of 79 COPD-associated genes. The third and fourth columns of the dataset list the corresponding protein IDs of the genes after querying their translated sequence against the UniProt database. The sixth and seventh columns contain information on the functional classification of genes based on Gene Ontology (GO) molecular function and biological process, respectively. The eighth and ninth columns contain information on the functional annotation of genes based on Clusters of Orthologous Groups of proteins (COG) analysis.

**Impact Statement**

Chronic obstructive pulmonary disease (COPD) is emerging as the third leading cause of human mortalities worldwide. Nontypeable *Haemophilus influenzae* (NTHi) is a major pathogen causing acute exacerbations resulting in diminished quality of life, hospitalization and increased risk of death in COPD patients. We analysed the core and accessory genome of 568 NTHi isolates, including 40 newly sequenced isolates, to genotypically distinguish between NTHi from COPD and other clinical phenotypes. This genome-wide analysis identified accessory gene content differences between COPD and non-COPD strains. It highlighted a set of virulence and metabolic functions that may be differentially required by COPD strains. This knowledge is important for developing improvements in the management of NTHi infections that can cause acute exacerbations in COPD patients.

## Introduction

While nontypeable *Haemophilus influenzae* (NTHi) is a common commensal of the human nasopharynx, this bacterium is also associated with a spectrum of diseases including otitis media and sinusitis, as well as hospital- and community-acquired pneumonia [1]. In addition, NTHi is the most common bacterial cause of chronic obstructive pulmonary disease (COPD) exacerbations [2–4]. NTHi has developed mechanisms to thrive in the hostile environment of different anatomical regions, such as the middle ear, upper and lower respiratory tracts, blood and the meninges [5]. Evolutionary and ecological forces drive bacteria to adapt and grow in different niches [6–9] by utilizing the basic nutrients available and resisting toxic products present in its environment [10]. This evolutionary adaptation typically involves two fundamental processes. The first is through mutations in genes, such as SNPs or nucleotide insertions/deletions, which can potentially alter the antigenicity of surface proteins or change the activity of enzymes and transport proteins [11]. A related mechanism is phase variation in which loci susceptible to hypermutation can undergo slipped-strand mispairing, due to changes in simple sequence repeats, which can rapidly modulate the expression level of genes [7, 12]. The second process is the acquisition of entirely new genetic sequences via horizontal gene transfer, which can undergo homologous or non-homologous recombination into the recipient genome [13]. During homologous recombination, a chromosomal fragment of a genome is replaced with a homologous sequence from another genome, whereas non-homologous recombination results in gain and loss of genetic material [14]. These processes can contribute to phenotypic changes including increased virulence and antibiotic resistance, and adaptations to the host microenvironments such as immune evasion and greater metabolic capacity [7, 8, 15–19].

Conventional typing methods such as multilocus sequence typing (MLST) cannot distinguish between commensal and pathogenic strains of NTHi [20–22]. Furthermore, a previous study by De Chiara and colleagues reported that phylogeny provides insufficient resolution to discriminate between strains isolated from different clinical sources based on an analysis of 97 NTHi isolates [21]. Here, we hypothesized that NTHi associated with COPD may exhibit genetically encoded functional variances when compared to the isolates from non-COPD clinical illnesses. Therefore, we performed an analysis on a larger set of 568 NTHi genomes, which included 40 Australian isolates that were newly sequenced in this study. Our analyses involved the application of pan-genome-wide association studies (pan-GWASs) of genes to determine whether NTHi from COPD could be discriminated from isolates from other types of clinical disease.

## Methods

### Bacterial strains collection, DNA extraction and genome sequencing

Forty NTHi isolates were retrieved from sputum samples of patients admitted to the Royal Hobart Hospital in Tasmania, Australia, from 2017 to 2018. Of these, 13 isolates were collected from COPD patients, with the remaining 27 collected from patients with other non-COPD disease presentations as shown in Dataset 1. DNA extraction and genome sequencing were performed using the protocol that has been described in detail in our previous methods paper [23]. Briefly, for DNA preparation, pure cultures of NTHi isolates grown on chocolate agar were suspended in PBS. Genomic DNA was extracted using a DNeasy blood and tissue kit (Qiagen) and was treated with RNase for the removal of RNA. The genomic DNA was further purified using a High Pure PCR template preparation kit (Roche), as described previously [23]. The genomic DNA library of these isolates was then prepared using the Nextera XT library preparation kit (Illumina) and loaded into an Illumina MiSeq v2 (2×150 bp paired-end reads) cartridge for sequencing using an Illumina MiSeq platform at La Trobe University, Australia.

In addition to the 40 Australian NTHi isolates that were sequenced, 528 publicly available NTHi genomes [7, 21, 24–29] were also downloaded from the National Center for Biotechnology Information (NCBI) (https://ftp.ncbi.nlm.nih.gov/genomes/refseq/bacteria/Haemophilus_influenzae/all_assembly_versions/) on September 10 2019 for analysis. The collection was composed of a heterogeneous group of isolates, in terms of geographical coverage. Based on the clinical source, the isolates were classified into two groups, COPD (*n*=373 isolates) and non-COPD (*n*=195 isolates).

### *De novo* genome assembly, annotation and pan-genome analysis

Raw fastq files generated from the Illumina sequencer were uploaded to the Galaxy web platform [30], and the St Petersburg genome assembler (SPAdes) tool [31] was used for the *de novo* assembly of the sequence reads [30]. The default settings for all parameters were used, except for the size of k-mers, which were manually chosen as 21, 33, 43, 53, 63 and 75. The quality of genome assembly was evaluated using the Quality Assessment Tool (quast) [32]. Coverage of the reference genome was determined by aligning all of the sequenced genomes to the reference complete genome of the strain 86–028 NP [24]. The 40 isolates sequenced in this study had on average 120 contigs (>500 bp), with a mean genome size of 1 906 568 bp, reference genome coverage of 87.3 % and a mean read depth of 118.8-fold.

The assembled fasta/fna files of the 40 Royal Hobart Hospital isolates and the 528 publicly available datasets were uploaded to the NeCTAR research cloud (http://cloud.nectar.org.au/) for subsequent analyses. We annotated relevant genomic features on the assembled/downloaded contigs using command line software tool Prokka version 1.12 with default parameters [33]. An *E* value threshold of 10^−6^ was used to determine the best match to known proteins in the databases, which included UniProt, Pfam and TIGRFAMs. If no matches were found, an ORF was labelled as a ‘hypothetical protein’.

The annotated genome assembly outputs from Prokka (in GFF3 format) were aligned to build large-scale pan-genomes using a rapid large-scale prokaryote pan-genome analysis pipeline, Roary version 3.12 [34]. An additional argument ‘-e -mafft’ was added to generate a multiFASTA alignment of core genes using mafft [35]. All other Roary parameters were used as default; minimum blastp identity of 90 %; Markov clustering algorithm inflation value of 1.5. The genes identified within the genome alignment were classified with respect to their presence among the isolates: core (≥95 %), shell (≥15 and<95 %) and unique or cloud genes (<15 %). The output files from Roary, *gene_presence_absence.csv* (Dataset 2) and *accessory_binary_genes.fa.newick*, were visualized using a Python script *roary_plots.py*, developed by Marco Galardini [36].

### *In silico* MLST

The draft and complete genomes of 568 NTHi isolates were genotyped based on the allelic profile of seven housekeeping genes (*adk*, *atpG*, *frdB*, *fucK*, *mdh*, *pgi* and *recA*) hosted at https://pubmlst.org/hinfluenzae/ [37]. Each isolate was assigned with a sequence type (ST) using *in silico* MLST typing [38]. A minimum spanning tree (MST) was then generated based on the MLST profiles using the goeBurst algorithm [39] and the tree was visualized using Phyloviz [40].

### Core-genome SNP (cgSNP) extraction and identification of genetic clusters

The core-genome alignment file was converted into a genlight object using the function *fasta2genlight* in the R package *adegenet* (version 2.1.1, RStudio version 1.0.143) [41]. The cgSNPs were extracted and analysed to determine clusters of genetically related isolates using the multivariate analysis method called discriminant analysis of principal components (DAPC) [42]. The cgSNPs raw data were initially transformed using principal component (PC) analysis, followed by the identification of genetic clusters using the *k-means* clustering algorithm. *k-means* determines a given number of groups (clusters) such that sequences belonging to the same cluster are more similar to each other than to sequences in other clusters. This was achieved using the function *find.clusters* with 150 PCs retained, accounting for >90 % of the sample variability. The optimal number of clusters was then inferred using the Bayesian information criterion as eight genetic clusters, which were then efficiently described using the *dapc* function with 60 PCs retained. An optimal number of PCs was chosen to avoid both extremes of a good model: underfitting and overfitting of the model. The trade-off between power of discrimination and overfitting can be measured by the α-score, which is the difference between reassignment probabilities for the true cluster and randomly permuted clusters [42]. We calculated the α-score for a range of retained PCs by implementing *a.score* and selected 60 as an optimal number of retained PCs at which the α-score was maximum at 0.7. The first three eigenvalues (discriminant functions) were then selected for visualization and interpretation.

In addition, we performed DAPC on the presence and absence profile of accessory genes to assess whether the composition of the accessory genome supports the partitioning of the collection into the identified clades. The same predefined clusters (clade I–VIII) identified by the core-genome-based *k-means* clustering were used to group the isolates. The accessory genome DAPC was carried out using the same methodology used for the core-gene DAPC except that the gene presence and absence matrix file was used instead of the core-genome alignment file. We retained the first 60 PCs and 3 eigenvalues to examine the genetic clusters. The mean α-score for 60 retained PCs was found to be 0.68.

### Phylogenetic analysis

cgSNPs were further utilized for performing phylogenetic analyses using megax software [43]. Evolutionary genetic distances between the strains were computed using the maximum composite likelihood method [44]. The evolutionary relationship was inferred using the neighbour-joining method [45]. Reliability of tree topology was tested using a bootstrap interior-branch test [46]. All the branches were supported by bootstrap values >90 %.

### Identification of COPD-associated genes

The distribution of COPD strains in the subpopulation (clades) of isolates was assessed. We also evaluated the genetic distinction between COPD and non-COPD strains using DAPC on cgSNPs and the presence/absence profile of accessory genes. A pan-GWAS approach was applied to determine whether any genes in the accessory genome were linked to a particular patho-phenotype including COPD using Scoary [47]. Scoary was implemented in Python using *gene_presence_absence.csv* (output from Roary) and *trait.csv* for the genotype and phenotype input files, respectively. Each candidate gene in the pan-genome was scored according to its apparent correlation to the clinical phenotype using a 2×2 contingency table of the presence/absence profile of each gene for the clinical phenotype. A Fisher’s exact test was performed on each gene in a population-agonistic manner. The Benjamini–Hochberg false discovery rate (FDR) adjustment was applied to correct for multiple comparisons [48]. The cut-off for a significant association was a *P* value lower than 0.05. Furthermore, for casual inference, a pairwise comparison algorithm was implemented that controls for spurious associations dependent on the population structure [49]. The phylogenetic tree calculated internally by Scoary from the Hamming distances in the genotype matrix was used for the pairwise comparisons [47]. The causal association was scored as significant when the *P* values for both the best and the worst pairings were lower than 0.05. Finally, an additional label-switching permutation was implemented by running pan-GWASs on randomly permutated phenotypic values between individuals for 1000 times and retaining the 5 % quantile, referred to as an empirical *P* value [50]. A minimum allele frequency threshold of 5 % was used so that genes present either in more than 28 isolates or in less than 540 isolates were included in the pan-GWAS analysis to avoid assigning too high importance to very rare genes/variants.

### Functional annotation and classification of candidate COPD genes

The nucleotide sequences of candidate genes were translated to their corresponding peptide sequences using EMBOSS Transeq (https://www.ebi.ac.uk/Tools/st/emboss_transeq/). The peptide sequences were then queried against the UniProt database using the basic local alignment search tool with an *E* threshold of 0.001 (https://www.uniprot.org/blast/). In order to better understand the underlying biological processes, we performed functional classification of the COPD-associated genes using the Gene List Analysis Tool that is accessed through the web-based panther version 14 classification system (http://pantherdb.org/) [51]. The complete Gene Ontology (GO) annotation system, which consists of three datasets, was used for mapping the functions of the genes of interest [52]. We classified our genes based on the GO molecular function and biological process.

## Results

### Global collection of 568 NTHi strains, including 40 newly sequenced Australian isolates

**Fig. 1.**
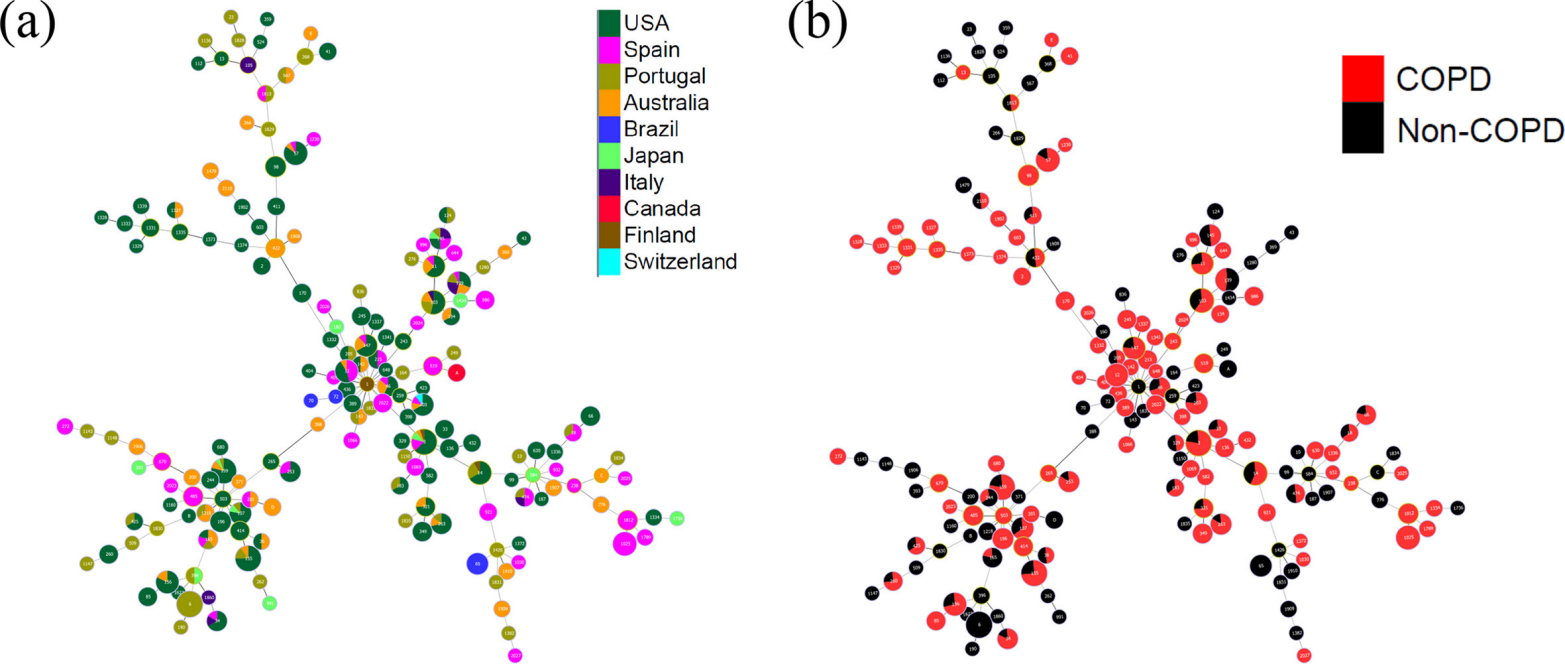
MST overview of 568 NTHi isolates based on MLST, i.e. allelic profiles of seven housekeeping genes present in the PubMLST database. This was generated using the goeBurst full MST algorithm and was visualized with Phyloviz 2. Each node is a ST, and it is coloured according to the (a) geographical and (b) clinical sources of the isolates. The size of each node is proportional to the number of isolates. The larger STs containing more than 10 NTHi isolates are labelled in-text. There was no absolute separation of the strains according to geography. (a) Based on the MLST profiling, strains from the same STs were common to a wide range of geographical locations. (b) COPD-associated isolates were scattered over the MST indicating a weak or no association between MLST genotype and COPD. Of 174 unique STs in our collection, COPD isolates were found in 70 STs.

For the initial assessment of the genetic diversity of the collection of NTHi isolates, we determined their ST using *in silico* MLST allelic profile. Based on the MLST profile, the 568 NTHi isolates were assigned to 174 unique STs [37]. Of which, 70 STs were associated with COPD, 34 STs contained both COPD and non-COPD strains, and the remaining 70 STs included non-COPD strains only (Fig. 1b). Sixty-one STs contained more than two NTHi isolates, of which twenty-seven STs were found to be associated with COPD (Fig. 1b). Some of the COPD-associated STs that contained five or more NTHi isolates were ST12 (*n*=11), ST98 (*n*=6), ST196 (*n*=7), ST349 (*n*=5), ST485 (*n*=5), ST1025 (*n*=13) and ST1812 (*n*=5).

A MST overview of all the isolates was generated by Phyloviz [40] (Fig. 1) using the goeBurst full MST algorithm based on MLST profile [39]. The MST overview was overlaid with the isolation data based on the geographical (Fig. 1a) and clinical (Fig. 1b) sources of the isolates. The distribution of the NTHi collection was uniform over the entire MST, in terms of geographical and clinical isolation (Fig. 1). There was no clustering specific to a geographical area. The USA, Spanish, Portuguese and Australian isolates were present in all groups and scattered throughout the entire tree. In addition, COPD isolates were also dispersed throughout the MST tree. By applying the traditional definition of clonal complex (CC) (genotypes which have allelic profiles that differ from the ST genotype at no more than one of the seven MLST loci, i.e. have at least six out of seven identical MLST genes) [53], we found 119 different CCs, of which 74 included a single ST. The largest CC consisted of only five STs and contained both COPD and non-COPD strains. Nine CCs included three or more STs; none of which were found to be specific to the COPD phenotype. This indicates a weak or no association between MLST genotype and COPD.

### Pan-genome analysis of 568 NTHi isolates

**Fig. 2.**
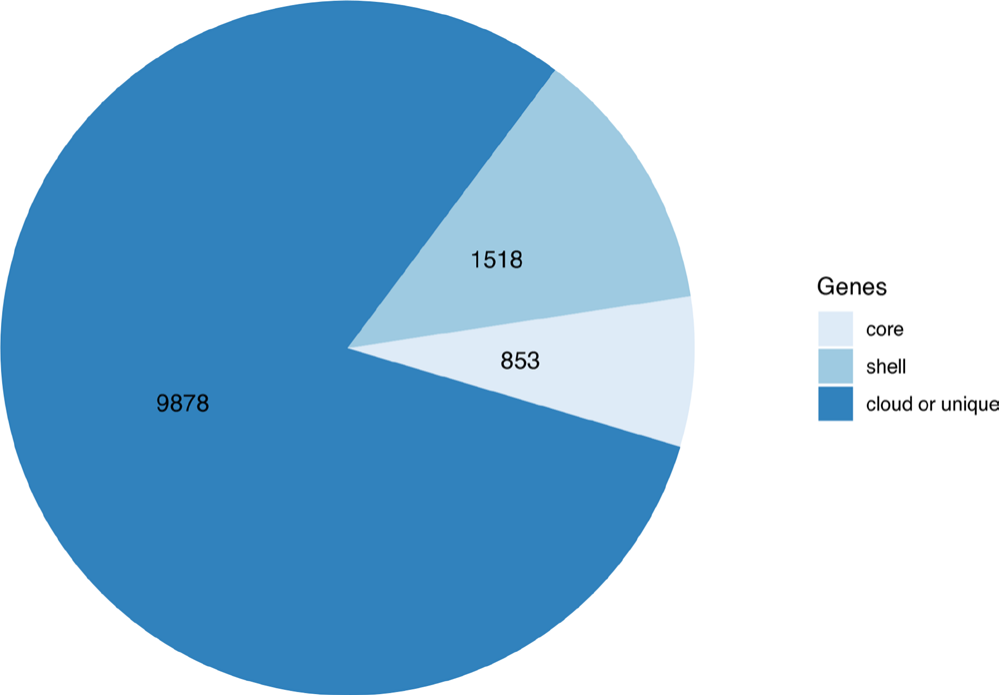
Distribution of genes present in the pan-genome of the NTHi collection (*n*=568), which was constructed using Roary version 3.12. Of 12 249 pan genes, the NTHi core-genome comprised 853 genes (present in at least 539 NTHi isolates). The remaining 11 396 genes of the accessory genome were further classified into the shell (*n*=1518 genes, present in less than 539 and at least 85 NTHi isolates) and cloud or unique (*n*=9878 genes, present in less than 85 NTHi isolates). On average, 47 % of each NTHi strain’s gene set belonged to the core pan-genome. The remaining 53 % of the strain’s gene set belonged to the larger accessory pan-genome. This accessory pan-genome encompassing a large repertoire of genes confers diversity and high levels of plasticity to the NTHi genome.

A pan-genome of 12 249 genes was generated from the 568 draft NTHi genomes, including the 40 isolates newly sequenced in this study (Fig. 2). The nucleotide sequences of non-redundant pan genes, as identified by Roary from theanalysis of 568 NTHi genomes, is included in the Pangene_sequence file. The core-genome was represented by 853 genes that were present in at least 539 isolates. The core genes accounted for approximately 47 % of the total number of genes (1821) present in the reference NTHi genome (86–028 NP) [24]. Whereas the core genome accounted for only 7% of the genes present in the pan-genome with the remaining 93% made up of the accessory pan-genome, which comprised the shell and cloud genes of the pan-genome (Fig. 2). The large accessory pan-genome confers diversity and high levels of genomic plasticity to NTHi. The majority of accessory pan genes (81 % of pan genes) were rare and strain-specific, found in less than 15% of the NTHi collection. The distribution of genes in the population had a characteristic U-shape, as reported in previous studies in *H. influenzae* and other bacterial species [21, 54, 55].

### Collection of 568 NTHi isolates exhibits a population structure defined by eight clades

**Fig. 3.**
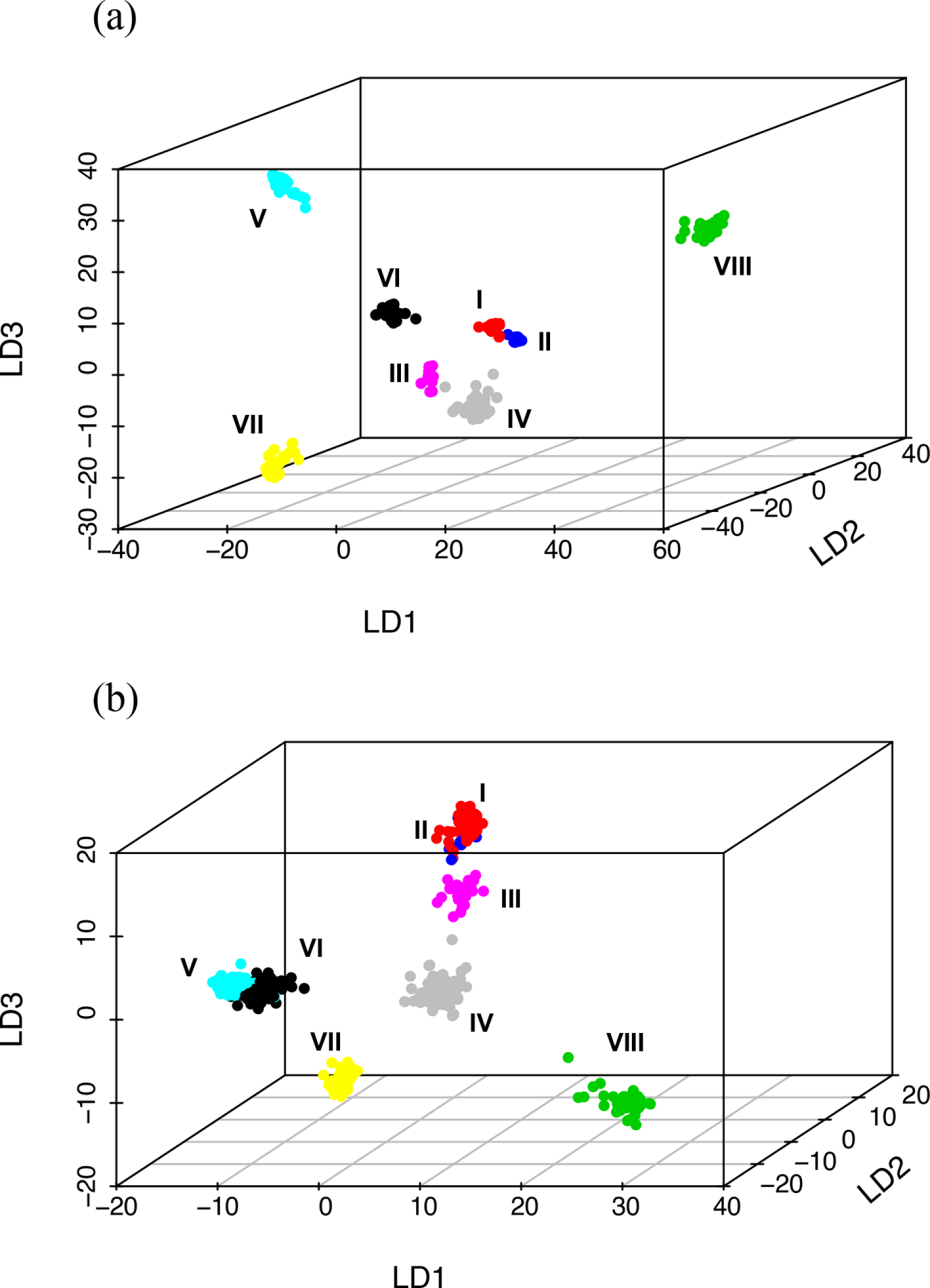
(a) 3D scatterplot of the cgSNP-based DAPC of NTHi isolates (*n*=568). DAPC resolved the NTHi isolates into eight clusters (clades). Clades I and II were closely related to each other, whereas all other clusters were distinctly separated. (b) The accessory-genes-based DAPC correlated with the cgSNP-based DAPC with a distinct separation of clades III, IV, VIII and VIII, and a close relationship between clades I and II. The only discrepancy was with the isolates of clades V and VI, which were clearly separated on cgSNPs DAPC, whereas they overlapped on the accessory-gene-content DAPC. Each dot is an isolate, coloured according to the classification into one of the eight clusters/clades as assigned by the cgSNP-based DAPC.

The core-genome alignment generated by concatenation of individual core-gene alignments was used to infer the population structure. We extracted 97 262 biallelic SNPs from the core-genome alignment of 696 234 bp. To this dataset, we applied the DAPC to infer the number of clusters of genetically related isolates. Bayesian information criterion supported the partitioning of this collection of isolates into eight clusters or clades that were clearly separated from each other, except for a small overlap between clusters I and II (Fig. 3a).

We also tested whether the presence/absence profile of accessory genes supported the partitioning of the collection into the predefined eight clades. For this, we performed a DAPC on the dispensable genes using predefined grouping as identified by the cgSNPs DAPC. Isolates of clades III, IV, VII and VIII were clearly separated from each other, while isolates in clades I and II were found to be more closely related, which was consistent with the cgSNPs DAPC (Fig. 3a, b). This suggests that the isolates in clades I and II are evolutionarily related and have a common ancestor. The composition of accessory-genome DAPC further showed a close association between clades V and VI, which was not observed with the cgSNPs DAPC (Fig. 3a, b). This indicates that a set of accessory genes that are shared between isolates of clades V and VI could have been either inherited from a common ancestor or acquired through horizontal gene transfer.

### Phylogeny separates isolates into groups that correlate with clades

**Fig. 4.**
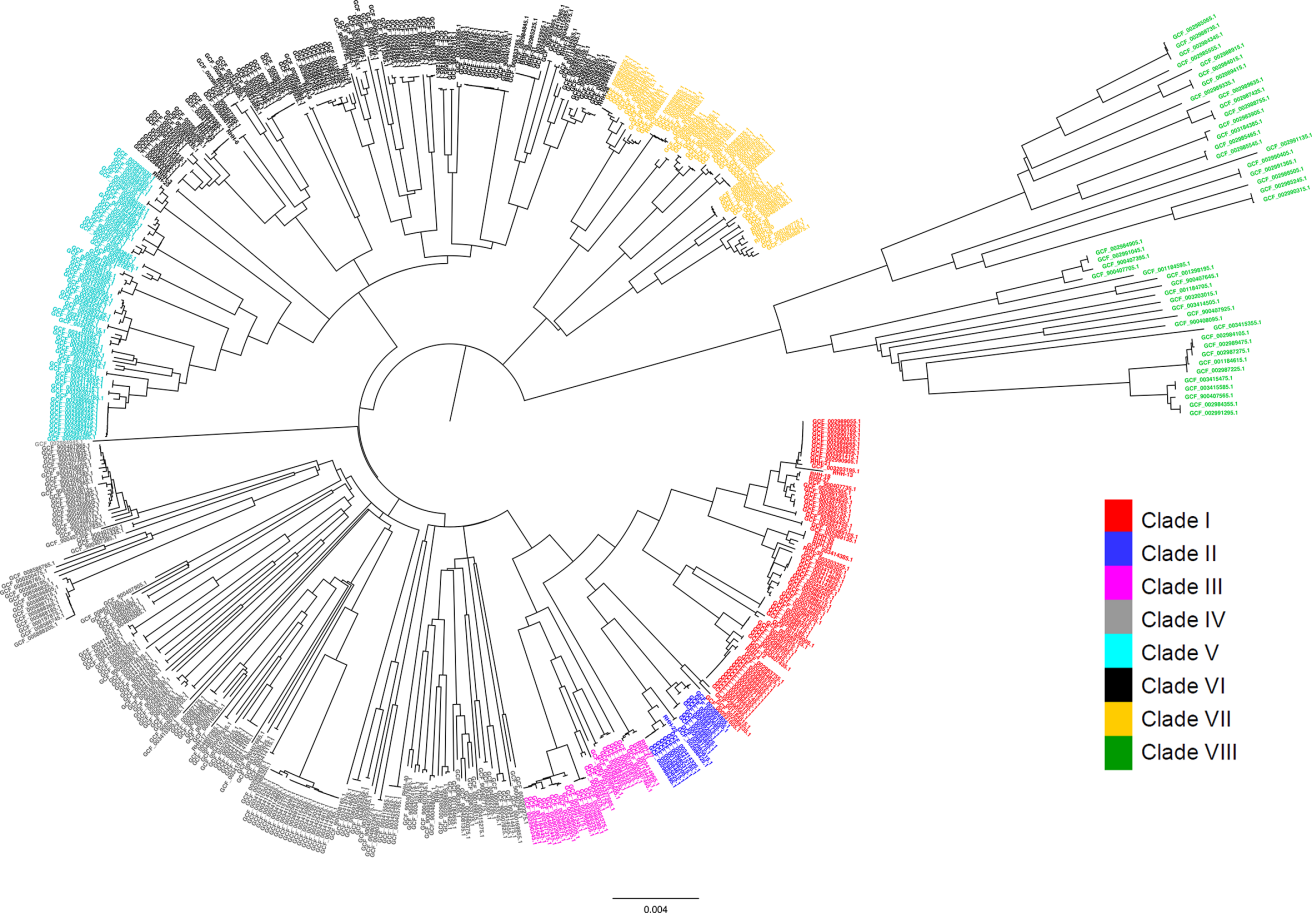
Neighbour-joining phylogenetic tree of 568 NTHi isolates, based on the cgSNPs. The evolutionary distances were computed using the maximum composite likelihood method. There was a total of 664 470 positions in the final dataset. Distinct sub-structuring of the NTHi population was evident with the cgSNP-based phylogeny. The phylogenetic analysis perfectly correlated with the DAPC-based classification that identified eight monophyletic clades. The close association between clades I and II, and between clades V and VI, as observed in accessory-genome-based DAPC, is consistent with the evolutionary relationship between them observed in the core-gene-sequence-based phylogeny obtained using the neighbour-joining maximum composite likelihood method. The tip labels are coloured according to the clades assigned by the cgSNP-based DAPC. Bar, number of base substitutions per site.

We then compared the clade partitioning to standard phylogenetic analyses. We built a neighbour-joining phylogenetic tree using the cgSNPs (Fig. 4). Molecular phylogenetic analysis correlated perfectly with the clade partitioning as defined by the population genetics, supporting the clonal nature of the NTHi population characterized by eight distinct lineages. Isolates of clades I and II were evolutionarily related, as suggested by the cgSNPs and the accessory gene DAPC. Consistent with the accessory gene DAPC, phylogenetic analysis showed a close relationship between clades V and VI.

The clade partitioning, as defined by population genetics and evolutionary relationship based on cgSNPs, partially correlated with the general clustering from the MLST profile (Fig. 1) as shown in Fig. S1 (available with the online version of this article). Clades I, II and III, and clades V and VI clustered together in the MST (Fig. S1), which correlated with the phylogenetic tree topology (Fig. 4). However, isolates of clades IV, VII and VIII did not form a distinct cluster, and were uniformly dispersed over the MST (Fig. S1). Furthermore, core-genome SNPs-based phylogeny did not result in separation of the strains according to geographical location of sample isolation (Fig. S2).

### Enrichment of genes in specific clades

Scoary, a pan-GWAS tool, was implemented to identify the genes that were enriched in a specific clade [47]. The FDR adjusted *P* value threshold of 0.001 was used, which identified 456, 167, 234, 532, 533, 599, 551 and 417 genes to be significantly overrepresented in clade I, II, III, IV, V, VI, VII and VIII, respectively (Dataset 3). Phylogenetically related clades shared a large proportion of clade-enriched genes. For example, clades I and II shared 29 % (141 out of 489 genes), and clades V and VI shared 32.6 % (278 out of 854 genes) of the genes enriched in the respective clades. Whereas, the evolutionarily distinct clades, such as clade VII and VIII, shared only 6.6 % (60 out of 908 genes) of the genes overrepresented in them.

### Composition of the accessory genome but not the distribution of cgSNPs separates COPD from non-COPD strains

**Fig. 5.**
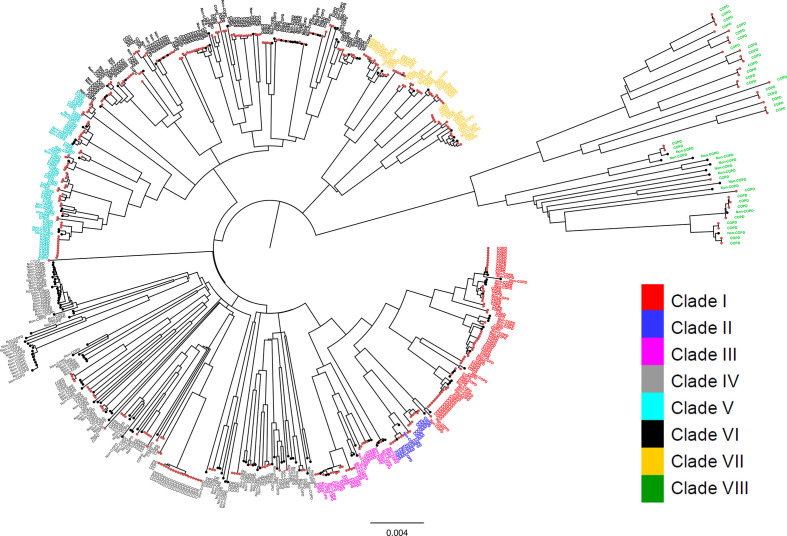
Distribution of 568 NTHi isolates over the cgSNP-based neighbour-joining phylogenetic tree, which is annotated with the clinical source of isolation of the samples as COPD or non-COPD. Each evolutionary clade includes both COPD and non-COPD strains. Branch tips representing COPD strains are highlighted in red, whilst those representing non-COPD strains are shown in black. The tip labels are coloured according to the clades (I to VIII) as assigned by the cgSNP-based DAPC. Bar, number of base substitutions per site.

**Fig. 6.**
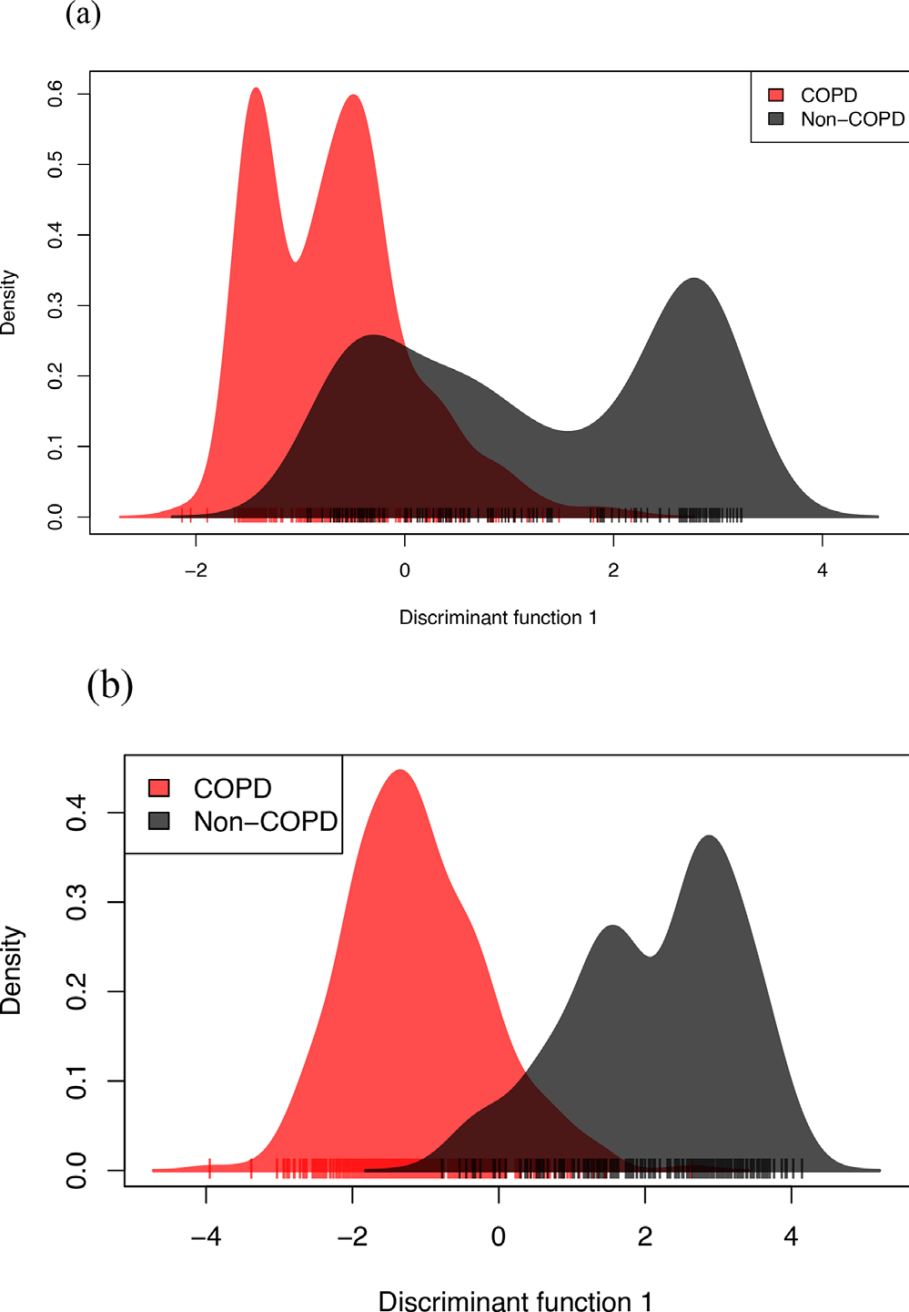
DAPC of 568 NTHi genomes from COPD and other disease isolates. (a) The first discriminant function of the retained PCs based on cgSNPs leaves substantial overlap between COPD and non-COPD strains, 356 NTHi isolates (231 COPD and 125 non-COPD strains) were in the overlapping region. (b) DAPC on the presence/absence profile of accessory genes clearly provides a higher level of separation of COPD from non-COPD strains with 226 NTHi isolates (119 COPD and 107 non-COPD strains) in the overlapping region. Composition of accessory genes, but not the distribution of polymorphic sites in the core-genome, discriminates COPD strains from other clinical phenotype strains of NTHi. Each line is an isolate. COPD and non-COPD isolates are coloured in red and black, respectively.

We then tested whether the 373 COPD strains could be associated with a particular clade(s). We overlaid the neighbour-joining phylogenetic tree with information on the clinical source of isolates as shown in Fig. 5. We found that COPD strains were distributed among all eight clades, with no absolute separation of the strains according to clinical source (Fig. 5). Expanding on our analysis, we next investigated whether the information on core-genome-wide polymorphic sites could be used to classify the collection into groups based on their clinical phenotypes. Discrimination analysis on cgSNPs showed significant overlap between COPD and non-COPD isolates, which is consistent with the previous results, suggesting poor resolution of cgSNPs for separating isolates according to their clinical source (Fig. 6a).

We then applied DAPC to the composition of the accessory genome, and found that, based on the first discriminant function, the presence/absence profile of dispensable genes could separate the COPD strains from the rest (Fig. 6b). The mean discriminant function separating the two populations was significantly different (*P*<0.0001, unpaired *t*-test with Welch’s correction). This result indicates that there is a difference in the composition of the accessory genome in NTHi that could potentially separate COPD strains from those associated with other clinical illnesses.

### Genome-wide association studies identified a set of accessory genes that are significantly associated with COPD strains

We determined the accessory genes in the bacterial dataset that were associated with a COPD phenotype. Scoary was used to screen the genes for association with COPD [47]. After the FDR correction, Scoary predicted 680 genes present in the NTHi pan-genome to be significantly enriched in NTHi collection from the COPD airways (Dataset 4). Out of the 680 genes, 226 genes were found to be significantly overrepresented in the COPD strains as compared to non-COPD strains (odds ratio ≥2, *P*<0.05) (Dataset 4).

Additionally, Scoary identified 145 (out of 680) genes to be significantly associated with the COPD phenotype (*P*<0.05) after pairwise comparison (Dataset 4). The other remaining 535 genes identified as significant prior to population-aware analysis were found to be lineage-specific effects upon inspection of the population distribution by the pairwise comparisons test. Furthermore, 1000 random permutations of the phenotype data were implemented, and the associated test statistic was calculated for each permutation. After the permutations, only 122 genes were found to have a significant association with the COPD phenotype (Dataset 4). Out of 122 significant hits, 86 hits were found to be different alleles (variants) of the same gene, one positively and one negatively associated with the COPD phenotype. The two alleles of these 43 genes were different enough to not be clustered as the same by Roary. Finally, there were 79 unique genes likely to play a role in COPD (Dataset 4).

### Functional classification of candidate COPD-associated genes

We next predicted the biological functions of the 79 unique COPD candidate genes. A total of 70 out of 79 genes mapped to UniProt proteins with a minimum identity threshold of 90 % (Dataset 5). Furthermore, the candidate genes were functionally annotated by assigning their encoded proteins to Clusters of Orthologous Groups of proteins (COGs) categories [56]. The COG analysis, however, did not result in an increase in confident functional prediction for the candidate genes as compared to the original annotations, i.e. 70 genes were assigned to known orthologous groups when the identity threshold was maintained at 90 % (Dataset 5).

Additionally, the functionally annotated genes that were associated with the COPD phenotype were classified based on their molecular functions and the biological processes they are involved in. Fifty-four and fifty-two of the seventy functionally annotated COPD-linked genes were assigned to GO molecular functions and biological processes, respectively (Dataset 5). A large number of these genes were found to be associated with transmembrane transporter activity (*n*=10), regulating the transport of inorganic phosphates, cations (Na^+^ and K^+^), metal ions such as copper and iron, lactate, dicarboxylate, carbohydrate, amino acids and proteins in and out of the bacterial cell (Table 1). Others were genes involved in cell redox homeostasis and cellular carbohydrate and protein metabolic processes, including the biosynthesis of aromatic and branched-chain amino acids. Consistent with previous studies [57, 58], a variant form of the IgA-peptidase-encoding gene has been found to be strongly associated with the COPD phenotype (odds ratio=4.4, *P*=0.0012). In addition, variants/alleles of genes encoding glycosyltransferases, such as *lex1* and *isgE*, and cytidylyltransferase-encoding *licC* that are involved in lipooligosaccharide (LOS) biosynthesis were also found to be associated with the COPD strains. With regard to other virulence genes such as *Haemophilus* adhesion and penetration protein (Hap), higher molecular weight proteins 1 and 2 (HMW1/2) and *H. influenzae* adhesin (Hia), they were found to be uniformly distributed among COPD and non-COPD strains.

**Table 1.** List of virulence and transformation competency associated genes that significantly correlated with COPD strains of NTHi

Gene	Gene name	OR	*P*	Pairwise *P*	Emp *P*	Function
*igA*	IgA1 protease autotransporter	4.5	0.0012	1.4×10^−7^– 1.53×10^−5^	0.0009	Virulence
*isgE*	*N*-Acetyl-glucosamine-transferase	5.5	0.0008	1.4×10^−6^– 6.6×10^−5^	0.0009	LOS synthesis
*lex1*	LOS biosynthesis protein lex-1	2.8	0.0274	3.6×10^−5^– 2.27×10^−2^	0.0009	LOS synthesis
*licC*	2-C-Methyl-d-erythritol 4-phosphate cytidylyltransferase	6.4	4.9×10^−9^	4.0×10^−8^– 2.3×10^−7^	0.0019	LOS synthesis
*pilA*	Type IV pilin subunit protein PilA	5.1	0.0002	3.1×10^−6^– 3.1×10^−6^	0.003	Adhesion; transformation
*tfoX*	DNA transformation protein	8.6	0.0003	3.7×10^−8^– 4.2×10^−7^	0.001	Transformation

OR, odds ratio; *P*, false rate discovery adjusted *P* value; Pairwise *P*, range of *P* values from the pairwise comparisons; Emp *P*, empirical *P* values after 1000 permutations.

*H. influenzae* is known to be naturally competent for transformation [59, 60]. *tfoX* (also called *sxy*) is a regulatory gene which is required for DNA uptake and transformation [61]. Its product, TfoX, interacts with cyclic-AMP receptor protein (CRP) and promotes the expression of genes of the competence regulon in *H. influenzae* [62, 63]. *H. influenzae* utilizes type IV pili for the transport of DNA across the membrane into the cytoplasm [64]. Variant forms of both *pilA* (encodes PilA, a major pilin subunit of type IV pili) [65] and *tfoX* are found to be associated with the COPD strains of NTHi (Table 1). RecJ is an exonuclease with 5′–3′ ssDNA-specific exonuclease activity that plays a crucial role in DNA repair and recombination pathways [66]. RecJ is associated with mismatch repair, and in addition, in conjunction with RecQ helicase, initiates recombination from a double-stranded break [67]. A variant of *recJ* has also been found to be associated with the COPD phenotype (odds ratio=2.5, *P*=0.019).

## Discussion

NTHi is associated with a wide range of diseases, including otitis media, meningitis and conjunctivitis, and is a major bacterial cause of exacerbations in COPD patients [3, 68–70]. The ability to predict a disease phenotype based on the genotype of a pathogen would be valuable in informing an appropriate prevention and treatment response. In some cases, phenotyping methods such as virulence factor profiling demonstrate clustering of the bacterial isolates into specific serotypes as in the case of *Streptococcus pneumoniae* [71]. While in others, MLST profiling correlates well with the disease induced by a pathogen, as in the case of *Enterococcus faecium *; for instance, a number of MLST STs, ST796, ST1421, ST233 and ST80, are associated with vancomycin resistance [72, 73]. In terms of infections with *H. influenzae *, capsulated strains have been reported to form serotype-specific clusters, in particular serotypes c, d, e and f that formed monophyletic clusters on a dendrogram reconstructed from the MLST allelic profile [21, 74]. However, the population of NTHi has been reported to be composed of a heterogenous group of isolates that form highly divergent clusters based on MLST [21]. Among the diseases caused by NTHi, Brazilian purpuric fever has been found to be caused by a well-defined NTHi clone (biogroup aegyptius) [75]; however, a correlation between genotypes of NTHi and COPD has not been established yet [21]. In this study, we analysed the whole-genome sequences of a large collection of NTHi strains that were isolated from different clinical sources to investigate a genetic basis of distinction between COPD and other phenotypic strains.

Our analyses indicate that conventional MLST typing exhibits low discriminatory power and is, thus, unsuitable for identifying COPD-specific clusters of NTHi. To increase the discriminatory power, we expanded the MLST scheme that comprises seven housekeeping genes and included 853 core genes in our analysis. We performed DAPC on cgSNPs, which grouped 568 NTHi isolates into distinct clades (Fig. 3a). Firstly, using this larger panel of genomes, the NTHi isolates resolved into eight clades compared to six clades in the previous analysis by De Chiara and colleagues in which 97 NTHi isolates were used [21]. NTHi isolates that fell within De Chiara’s clade I and V further separated into two distinct clusters each; whereas, the other clades (II, III, IV and VI) did not show substructure with the analysis of 568 NTHi isolates. Each of the eight clades contained NTHi strains from diverse disease phenotypes. Moreover, phylogeny derived from an analysis of cgSNPs did not differentiate NTHi isolates based on their clinical source which is consistent with the study conducted by De Chiara *et al.* and a later study by Pettigrew and colleagues that analysed a collection of 403 NTHi genome sequences [7, 21]. The finding that NTHi strains with highly similar core-genome sequences can cause a wide range of diseases suggests that non-core accessory genes may to a large extent be responsible for the different disease phenotypes that result from NTHi infection, as has been observed in other pathogenic bacteria such as *Clostridium difficile* [76].

The eight distinct NTHi clades supported by population genetics correlated perfectly with molecular phylogenetic analysis based on the cgSNPs (Figs 3a and 4). Analysis of the composition of the accessory genome further correlated with phylogeny (Figs 3b and 4). This consistency highlights the clonal nature of the NTHi population. We also identified the genes that were enriched (over-represented) in each clade (Dataset 3) and found a set of clade-enriched genes distributed over the evolutionarily distant clades. For example, out of 456, 551 and 417 genes that were specifically overrepresented in clades I, VII and VIII, respectively, (FDR adjusted *P* value <0.001), 11 genes were found to be common in these phylogenetically distinct clusters. This underscores the role of horizontal gene transfer prevalent in the NTHi population that accounts for genetic diversity among the species [77].

We then applied discriminant analysis on the composition of accessory genes, which demonstrated a clear separation between strains associated with COPD and other clinical illnesses. Moreover, using a pan-GWAS approach, we identified a subset of NTHi accessory genes associated with the COPD phenotype. Some key COPD-associated genes likely to be involved in pathogenesis are listed in Table 1. NTHi utilizes cells surface structures to interact with host cells and pave the way for colonization and invasion [78]. We identified a variant form of *pilA*, encoding type IV pilin subunit protein, and LOS biosynthetic genes (*licC, lex1* and *isgE*) as being associated with the COPD strains. PilA and LOS have been demonstrated to play an important role in biofilm formation and colonization of the respiratory tract [79, 80]. Evasion of host immune defence is another probable mechanism by which NTHi strains thrive in the COPD airways. We found an association of the gene encoding IgA1 protease, which cleaves immunoglobulin A (IgA), with COPD isolates of NTHi (Table 1). IgA1 protease has previously been reported to be important to *H. influenzae* in the lower airways of COPD patients [81].

Pettigrew’s and Molere’s groups recently investigated large prospectively collected NTHi genomes to give insight into molecular changes during persistence in the COPD lung [7, 25]. They found genetic changes in multiple genes that regulate expression of virulence functions, such as adherence, nutrient uptake and immune evasion, which are likely to be involved in NTHi survival in the COPD lung. This suggests that in comparison to other ecological niches, such as the middle ear, sinuses, eye, meninges and the upper respiratory tract, NTHi in COPD airways are exposed to different microenvironments defined by distinct nutrient availability, pH, oxidizing potential and/or immune response. NTHi, therefore, exhibits genomic changes which appear to aid survival and adaptation in the hostile environment of COPD airways. Consistent with these findings, we found that COPD isolates of NTHi encoded different metabolic activities compared to strains associated with other clinical phenotypes. This suggests that metabolic capacity, in part, plays an important role in enabling NTHi to contribute to COPD pathogenesis and further supports the concept of nutritional virulence as an important determinant of pathogenic capability in NTHi [82].

In conclusion, our study indicates that the virulence and survival of NTHi in COPD is influenced by genes outside of the core-genome. The set of accessory genes associated with COPD strains may assist in successfully establishing a niche in COPD airways through acquisition of nutrients, evasion of the immune response, and enhancement of adhesion and colonization of airways. In addition, the presence of competence and recombination genes may enable NTHi strains to acquire genes that confer a competitive advantage in the COPD airways. Further work will examine how our finding that COPD strains of NTHi possess a distinct gene content could be translated into improvements in the management of NTHi infections in COPD.
