# Corrigendum: Whole-genome analyses reveal gene content differences between nontypeable Haemophilus influenzae isolates from chronic obstructive pulmonary disease compared to other clinical phenotypes

**DOI:** 10.1099/mgen.0.000665

**Published:** 2021-10-04

**Authors:** Rajendra K. C., Kelvin W. C. Leong, Nicholas M. Harkness, Julia Lachowicz, Sanjay S. Gautam, Louise A. Cooley, Belinda McEwan, Steve Petrovski, Gunasegaran Karupiah, Ronan F. O'Toole

**Affiliations:** ^1^​ Tasmanian School of Medicine, College of Health and Medicine, University of Tasmania, Tasmania, Australia; ^2^​ Department of Pharmacy and Biomedical Sciences, School of Molecular Sciences, College of Science, Health and Engineering, La Trobe University, Victoria, Australia; ^3^​ Department of Respiratory and Sleep Medicine, Royal Hobart Hospital, Tasmania, Australia; ^4^​ Department of Microbiology and Infectious Diseases, Royal Hobart Hospital, Tasmania, Australia; ^5^​ Department of Physiology, Anatomy and Microbiology, School of Life Sciences, La Trobe University, Victoria, Australia

In the published version of Supplementary Dataset 1 associated with this article and hosted on figshare at https://doi.org/10.6084/m9.figshare.12545957.v2, there was an error in the listing of the NTHi isolate on line 551. ‘RHH-3’ should have read ‘RHH-15’.

This has now been corrected in the updated Supplementary Dataset which can be found on figshare at: https://figshare.com/s/8d95658f8bda7eed27c4.

The authors regret any inconvenience caused.

